# Celebrating 25 Years of MicroRNA Research: From Discovery to Clinical Application

**DOI:** 10.3390/ijms20081987

**Published:** 2019-04-23

**Authors:** Lorenzo F. Sempere

**Affiliations:** Department of Radiology, Precision Health Program, Michigan State University, East Lansing, MI 48824, USA; semperel@msu.edu; Tel.: +1(517)355-3982

In 1993, the Ambros lab reported the cloning and developmental function of lin-4, the first microRNA [1]. This short non-coding RNA was regarded as an oddity of the mighty little roundworm *Caenorhabditis elegans*, controlling gene expression by binding to partially complementary sites on the 3′ UTR of target mRNAs and inhibiting translation [1,2,3]. The discovery of let-7, also in *C. elegans*, reinforced this molecular oddity and highlighted the power of forward genetics in this model organism [4]. These two small temporal RNAs control developmental timing of larval transition in *C. elegans* and other *Caenorhabditis* species [5]. This restricted classification was short-lived as the evolutionary conservation of let-7 [6] and the shared enzymatic machinery for processing of endogenous short non-coding RNAs and exogenous double-stranded RNA substrates for RNA interference [7] strongly suggested a broadly co-opted regulatory mechanism of short non-coding RNAs in animal evolution. Indeed, let-7 and miR-125 (lin-4 paralog) family members have been implicated in temporal identity in the fly brain, and are likely involved in temporal cell fate decision in vertebrates [5]. Over the last 20 years, microRNAs have been identified in many animal species. Some of these microRNAs are phylum-, order-, genus-, or even species-specific [8,9,10]. The size of the microRNome and complexity of animal body plans and organ systems suggest a role of microRNAs in cell fate determination and differentiation [8,9]. More than 2000 sequences have been proposed to represent unique microRNA genes in humans with an increasing number of mechanistic roles in developmental, physiological, and pathological processes [10,11].

MicroRNA are short non-coding regulatory RNAs. The mature and biologically active form is about 18–25 nucleotides long. This mature sequence binds to and guides an Ago-containing, RNA-induced, multi-protein silencing complex to partially complementary sites on the mRNA of target genes [12]. Due to this partial complementarity, a single microRNA can regulate the expression of many, if not hundreds, of target genes. Thus, dysregulation of a few key microRNAs can have a profound global effect on gene expression and molecular programs of a cell. Conversely, restoration of baseline microRNA activity can also have profound effects to reverse a pathological process [12,13]. This great potential for clinical intervention captured the interest and imagination of researchers in many fields. However, very few fields have been as prolific as the field of cancer research. This is largely due to early studies by Carlo Croce and colleagues that linked microRNAs with cancer just a couple of years after the discovery of let-7. In 2002, Calin et al. showed that chromosomal deletion of miR-15 and miR-16 was a frequent event in B-cell chronic lymphocytic leukemia [14]. Soon after, Calin et al. also made the tantalizing observation that many microRNA loci are located at fragile sites, breakpoint regions, or frequently altered regions (e.g., deletion or amplification) in the cancer genome [15]. These seminal papers, along with a technological explosion of high-throughput detection platforms, led several groups to extensively profile microRNA expression in healthy and tumor tissues. Altered microRNA expression has been associated with diagnostic and prognostic indicators in many cancer types. Different mechanisms have been reported for this dysregulation, including chromosomal deletion or amplification of a microRNA gene, epigenetic and transcriptional regulation, and mutation in the enzymes responsible for microRNA processing, export, or silencing [12,13]. Despite the overwhelming number of diagnostic and prognostic studies, the current impact of microRNA-based assays is very limited in clinical practice. Some microRNA-based assays have reached the clinic in the form of laboratory developed tests, and several on-going clinical trials propose the use of microRNAs as biomarkers for early disease detection, guiding treatment selection, monitoring disease progression, or other specific clinical endpoints.

This Special Issue celebrates the 25th anniversary of the discovery of the first microRNA. It provides but a glimpse of the large body of literature of microRNA biology in cancer research. This Special Issue contains four original research studies [16,17,18,19] and four review papers [20,21,22,23] with a disease focus on specific hematologic or solid tumors. Collectively, these papers highlight state-of-the-art approaches and methodologies for microRNA detection in tissue, blood, and other body fluids for biomarker applications from early cancer detection to prognosis and treatment response. These papers also address some of the challenges for clinical implementation. Pezzuto et al. provide a comprehensive review of blood-based detection of cell-free DNA and microRNAs for early detection of hepatitis viruses–related liver cancer [21]. Chang et al. report on a plasma-based 3-microRNA signature for early detection of oral cancer [17]. Konstantinell et al. provide a comprehensive review of extracellular vesicle-based and tissue-based detection of microRNA for diagnostic and prognostic applications in Merkel cell carcinoma [22]. de Oliveira et al. provide a comprehensive review of tissue-based and cell-based detection of microRNAs for diagnostic and prognostic applications in childhood hematological cancers [23]. Drobna et al. describe a methodological advancement of most suitable endogenous microRNA controls for cell-based microRNA detection in T-cell acute lymphoblastic leukemia [18]. Hibner et al. provide a comprehensive review [20], while Moller et al. [19] and Ulivi et al. [16] present specific studies of microRNA biomarkers for diagnostic and prognostic applications in colorectal cancer. Below is a brief summary and highlights of each of these articles.

Liver cancer is the third leading cause of cancer-related death in the world. Hepatocellular carcinoma (HCC) represents 85% of all liver cancer cases and generally has a poor prognosis due to late presentation. Chronic infection of Hepatitis B or Hepatitis C viruses are a major risk factor for development of HCC. Pezzuto et al. review blood-based detection assays that would complement and improve current diagnostic tools based on various imaging modalities and plasma levels of alpha-fetoprotein [21]. Pezzuto et al. describe the utility of detecting circulating cell-free DNA, and cell-free or extracellular vesicle-loaded microRNAs and long non-coding RNAs. While DNA mutations or altered DNA methylation pattern in circulating DNA or altered circulating microRNA levels can detect HCC tumors, altered levels of some microRNAs such as miR-122 can reflect more closely the effect of viral infection on malignant transformation of hepatocytes. Of the discussed blood-based markers, Pezzuto et al. highlight altered circulating levels of miR-122 and let-7, and RASSF1A hypermethylation in circulating free-DNA as the most promising biomarkers for diagnostic application. As in HCC, patients that present with late stage oral cancer, which can be as high as 50% of all cases in some countries, have a much worse prognosis than early stage cases. Oral squamous cell carcinoma (OSCC) is the most common type of oral cancer. Prevalence and risk factors vary with geographic location. In South and Southeast Asia and Taiwan, betel quid chewing is a major risk factor. Chang et al. performed RNA sequencing from plasma of healthy controls, early stage patients with oral leukoplakia (OL, precursor lesion associated with OSCC progression) and late stage patients diagnosed with OSCC to identify differentially expressed microRNAs. Selected microRNAs were further studied in a training set of 72 samples and validated in an independent set of 178 samples of Taiwanese patients. From these analyses, Chang et al. identified a 3-microRNA signature (miR-150-5p, miR-222-3p, miR-423-5p) that could accurately separate OL from OSCC cases and may have clinical utility for early detection of OSCC. As in HCC, viral infection is a major risk factor in Merkel cell carcinoma (MCC), which is a rare but aggressive type of skin cancer. About 80% of MCC tumors are infected with viral DNA of Merkel cell polyomavirus. Konstantinell et al. provide a comprehensive review on the host and viral microRNA expression in MCC tissue samples and present their original data profiling microRNA expression in extracellular vesicles secreted by MCC cell lines [22].

MicroRNA expression and function have been extensively studied during normal hematopoiesis and in several hematological malignancies, including different types of leukemias and lymphomas. Childhood leukemia and lymphomas have certain features that distinguish them from adult counterpart conditions such as mutational spectrum, cell of origin and location, and cellular context and microenvironment. de Oliveira et al. provide a comprehensive review of microRNA dysregulation in childhood leukemia and lymphomas and contrast their differences with adult counterpart conditions [23]. Childhood leukemias and lymphomas represent about 30% and 15% of all pediatric tumors, respectively. de Oliveira et al. systematically review the literature for each leukemia and lymphoma cancer types, with an emphasis on major types, including acute lymphoblastic leukemia (ALL), acute myeloid leukemia and Burkitt lymphoma. In addition to detailed text descriptions, de Oliveira’s colorful figures provide informative and concise graphical summaries of microRNA dysregulation associated with diagnostically and therapeutically relevant criteria in each condition such as chromosomal rearrangement and treatment response to a specific drug regimen. While de Oliveira et al. focus their review on microRNA detection in cancer cells or tumor tissues and its diagnostic and prognostic implications, the authors also discuss emerging studies on circulating microRNAs in acute lymphoblastic leukemia and other conditions.

Reliable endogenous controls for normalization of microRNA expression in cells or tissues or for circulating microRNA levels are an important consideration to maximize accuracy of biomarker readout. Identifying such controls is technically challenging because microRNA expression is cell type-, context-, and disease-dependent. Drobna et al. describe a strategy to identify endogenous microRNA controls for adult T-cell ALL using a reverse-transcription quantitative PCR (RT-qPCR) assay [18]. Drobna et al. performed RNA sequencing analysis on sorted T cells from 34 T-cell ALL cases and from bone marrow of five healthy controls. Using an algorithm to identify microRNA with stable expression across the samples, the authors selected 10 microRNAs for further evaluation by RT-qPCR assay; most of these 10 microRNAs had been previously suggested to serve as appropriate controls in other tissue types or disease conditions. Drobna et al. propose three microRNAs (let-7a-5p, miR-16-5p, miR-25-3p) as optimal endogenous controls for evaluation of T-cell ALL samples.

Colorectal cancer (CRC) is the second leading cause of cancer-related death in the world. With over a million new cases and over half a million deaths yearly, clinical management of colorectal cancer is an important and worldwide health problem. Hibner et al. provide a comprehensive review of blood-based and tissue-based studies that utilize a single microRNA or a microRNA signature to find association with diagnostic and prognostic indicators in CRC [20]. Hibner et al. devote individual sections to microRNAs frequently associated with CRC in multiple independent studies, including miR-21, miR-29b, miR-34a, and miR-155. Although these sections focus on diagnostic and prognostic applications, Hibner et al. also report on specific targets of these miRNAs and their potential application for therapeutic intervention. RT-qPCR assay is the preferred method for miRNA expression analysis in most of these CRC studies as well as in other studies described above. The study by Ulivi et al. exemplifies the robustness of RT-qPCR for detection of circulating miRNAs [16]. Ulivi et al. analyzed plasma levels of miR-20b, miR-29b, and miR-155 in a cohort of 52 metastatic CRC cases treated with bevacizumab-containing chemotherapy regimen. Higher circulating levels of these three microRNAs in plasma collected before treatment were associated with longer progression-free and overall survival. The authors only analyzed these microRNAs individually, thus it will be interesting to see if this 3-miRNA signature would provide a stronger prognostic signal. Intriguingly, comparison of circulating levels of these microRNAs before treatment and after 1 month of treatment showed that cases with increased levels of miR-155 after treatment are associated with shorter progression-free and overall survival. The authors discuss mechanisms for the timing and opposite outcome based on circulating miR-155 levels. However, RT-qPCR assay has limitations and is technically challenged to apply for miRNA detection in specific cell types that compose the tumor mass. Møller et al. 2019 describe elegant and robust methodology for in situ co-detection of microRNAs, mRNAs, and non-coding RNA molecules in tumor tissues, combining locked nucleic acid chemistry for microRNA probes and RNAscope^®^ technology for mRNA probes [19]. This in situ co-detection assay enables characterization of RNA expression at single cell resolution providing biologically relevant information of the cell type(s) that present with altered regulation of miR-21 expression in a particular tumor. Previous studies by this group and others have shown that miR-21 expression is predominantly upregulated and carries prognostic value in cancer-associated fibroblasts (CAFs) more than in cancer cells (reviewed in Hibner et al. [20]). Curiously, Møller et al. [19] report upregulation of miR-21 in a discrete set of cancer cells, budding cells, in addition to CAFs in a panel of colorectal cancer cases. Budding cancer cells are single or a small cluster of cells at the invading front that pinched off or detach from the main tumor mass. Co-detection of miR-21 and TNF-α mRNA expression did not indicate a regulatory relation between this pro-fibrotic and pro-survival microRNA and this pro-inflammatory cytokine in budding cancer cells. Nonetheless, upregulation of miR-21 expression suggests a potential role in the survival and/or migration of budding cells.

I would like to thank the authors for their valuable contributions to this Special Issue. I also would like to thank editorial staff members, especially Meredith Liu, and anonymous reviewers who improved the presentation and content of this Special Issue. I hope readers find this Special Issue an accessible reference to keep abreast of recent findings, methodologies, and approaches related to microRNA biology in cancer research and its potential applications in cancer medicine.
